# Prevalence of Insomnia among Pancreatic Cancer Patients following Pancreaticoduodenectomy

**DOI:** 10.1155/2021/5535220

**Published:** 2021-05-04

**Authors:** Sabine Chalhoub, Marita Yaghi, Natasha Ard, Mariam Kanso, Jad Allam, Mohamad Khalife, Rola F. Jaafar, Walid Faraj

**Affiliations:** Department of General Surgery, American University of Beirut Medical Center, Beirut, Lebanon

## Abstract

**Introduction:**

Sleep disturbances are more common in cancer patients than in the general population; however, there is limited research pertaining to the occurrence of such disturbances that subsequently impact patients' quality of life. The aim of our study is to describe the prevalence of insomnia among pancreatic cancer patients who have recently undergone recent pancreaticoduodenectomy.

**Methods:**

We performed a 6-year (2014-2020) retrospective cohort analysis of all adult patients aged 18 and above with pancreatic cancer who underwent pancreaticoduodenectomy at our institution. Insomnia was characterized using the Pittsburgh Sleep Quality Index (PSQI). Symptoms of insomnia and the impact caused by these symptoms on daily lives were assessed with the Insomnia Severity Index (ISI), and patients were divided into mild insomnia (ISI 8–14) or moderate to severe insomnia (ISI ≥ 15).

**Results:**

Out of forty patients with pancreatic cancer that have undergone pancreaticoduodenectomy, 19 (47.2%) reported that their sleep disturbances had a significant effect on their quality of life. A total of 22 (55.0%) patients reported insomnia, with 63.2% reporting mild insomnia. Chemotherapy was found to significantly increase the risk of moderate to severe insomnia. The mean ISI score was 7.2 ± 6.9, and the mean PSQI score was 7.0 ± 5.1. ISI and PSQI showed a strong positive correlation (*r* = 0.78, *p* < 0.01).

**Conclusion:**

Sleep disturbances such as insomnia following pancreatic cancer surgery are highly prevalent. Treating physicians and surgeons should recognize and routinely screen for sleep disorders through the management of a multidisciplinary team in order to alleviate some of the burden on the patients' mental well-being.

## 1. Introduction

Sleep and health are strongly related. Sleep quality is positively related to physical and mental restoration [1], whereas sleep deprivation has been consistently associated with a decline in physical and cognitive [2] function. Insomnia is defined as the subjective perception of difficulty with sleep initiation, duration, consolidation, or quality, which occurs despite adequate opportunity for sleep and results in some form of daytime impairment [3]. Chronic insomnia has more specific diagnostic criteria set forth by the Diagnostic and Statistical Manual of Mental Disorders, Fifth Edition (DSM-V) stating that symptoms of insomnia need to be present for at least 3 times a week for a period of at least 3 months and to not be explained by other sleep, medical, or mental disorders [4]. Insomnia negatively impacts decision-making capacity and instigates issues in work performance and relationships, leading to an overall decline in the quality of life [5, 6].

Insomnia is more frequent in cancer patients than in the general population, where up to 50% of recently diagnosed or treated cancer patients report sleeping difficulties [7], possibly due to the drugs and treatments received, among other causes. Surgery, also a reported major cause of sleep disturbances, has a more pronounced effect in cancer patients [8]. Despite significant prevalence and consequences, cancer-related insomnia has been given little attention by researchers and clinicians [7].

Cancer-specific insomnia studies are scarce, more so in pancreatic cancer patients. The aim of this study is to examine the prevalence of insomnia in patients with pancreatic cancer who have undergone recent pancreaticoduodenectomy.

## 2. Patients and Methods

We conducted a 6-year (2014 to 2020) retrospective cohort study of all adult patients with pancreatic cancer who presented to our center and underwent pancreaticoduodenectomy. A study approval was obtained from the American University of Beirut Institutional Review Board. All eligible patients were approached for the consenting process, and those who agreed to participate in the study were included in the analysis.

### 2.1. Study Population: Exclusion Criteria

All patients who presented with unstable psychiatric illness, with noncancer medical illness, or with established sleeping disorders prior to the study, were excluded. Patients taking medications known to influence sleep or involved in a night shift-based employment schedule were also excluded. Patients unwilling to consent or unable to answer questions without family historians were also excluded.

### 2.2. Study Protocol

All patients with pancreatic cancer who underwent pancreaticoduodenectomy were identified through the medical records department at the hospital, and all of those who were eligible were identified and approached by the investigators. The study protocol, along with the benefits and risks, was explained to every eligible patient. A phone survey that measured subjective sleep quality and symptoms of insomnia was used after obtainment of the patient's oral consent. Data points regarding demographics were collected through a review of electronic medical records.

### 2.3. Data Points

#### 2.3.1. Sleep Quality

The Pittsburgh Sleep Quality Index (PSQI), which is a self-report survey that assesses sleep quality over a 1-month time interval, was used to assess subjective sleep quality in our study. The PSQI consists of 19 individual items, and it measures several different aspects of sleep, offering seven component scores and one composite score. The component scores consist of subjective sleep quality, sleep latency, sleep duration, habitual sleep efficiency, sleep disturbances, use of sleeping medication, and daytime dysfunction [9]. A total score, ranging from 0 to 21, is obtained by adding the seven component scores. A score > 5 suggests poor sleep quality.

#### 2.3.2. Insomnia Symptoms and Quality of Life

The Insomnia Severity Index (ISI), which is a brief self-report instrument measuring the patient's perception of both nocturnal and diurnal symptoms of insomnia, was used in our study. The ISI comprises seven items assessing the perceived severity of difficulties initiating sleep, staying asleep, and early morning awakenings, satisfaction with the current sleep pattern, interference with daily functioning, noticeability of impairment attributed to the sleep problem, and degree of distress, or concern caused by the sleep problem [10]. A 5-point Likert scale is used to rate each item, yielding a total score ranging from 0 to 28. The total score is interpreted as follows: absence of insomnia (0–7), subthreshold insomnia (8–14), moderate insomnia (15–21), and severe insomnia (22–28).

### 2.4. Outcomes

Our primary outcome measures the prevalence of insomnia in patients with pancreatic cancer who have undergone pancreaticoduodenectomy.

### 2.5. Patient Stratification

Patients were grouped based on ISI and PSQI scores. Patients who suffered from subjective decrease in quality of sleep were defined by a PSQI score of more than 5. Patients who suffered from mild clinical insomnia were defined by an ISI score of 8 to 14, and patients who suffered from moderate or severe clinical insomnia were defined by an ISI score of 15 or above.

### 2.6. Statistical Analysis

We performed descriptive statistics. Continuous parametric data were summarized using mean ± standard deviation. Continuous nonparametric data were summarized using a median with an interquartile range. Categorical data were reported using proportions and percentages. To analyze the differences between the mild insomnia and moderate to severe insomnia groups on a univariate level, we used a chi-square test for categorical variables, the Mann-Whitney *U* test for continuous nonparametric data, and independent Student's *t*-test for continuous parametric data. To analyze the bivariate correlation between clinical insomnia and quality of sleep, we used Pearson's parametric test for normally distributed continuous variables.

We considered a *p* value of less than 0.05 (*p* < 0.05) as statistically significant. All statistical analyses were carried out using the Statistical Package for the Social Sciences (SPSS, version 26; SPSS, Inc., Armonk, NY).

## 3. Results

### 3.1. Patient Characteristics

Forty patients were enrolled in our study. All patients completed the interview required by the study, and none were subsequently excluded from our final analysis. The mean age was 66.4 ± 10.1 years at the time of questionnaire administration. 67.5% of patients were male and 32.5% were females. Most patients had a mildly overweight BMI of 25.7 ± 4.3. Almost half of the patients (45.0%) reported intake of caffeine and smoking, 20.0% reported alcohol consumption, and 55.0% engaged in physical activity. The most common comorbidities were diabetes (42.5%) and cardiovascular disease (52.5%). Less common comorbidities were chronic obstructive pulmonary disease (5.0%) and chronic kidney disease (4.0%) (Table 1).

All patients had undergone surgery for their pancreatic cancer within 7 months of questionnaire administration. Tumor characteristics such as tumor grade and stage, tumor size, treatment regimen, and all-cause mortality are additionally reported in Table 1.

### 3.2. Insomnia Scores

**Figure 1:**
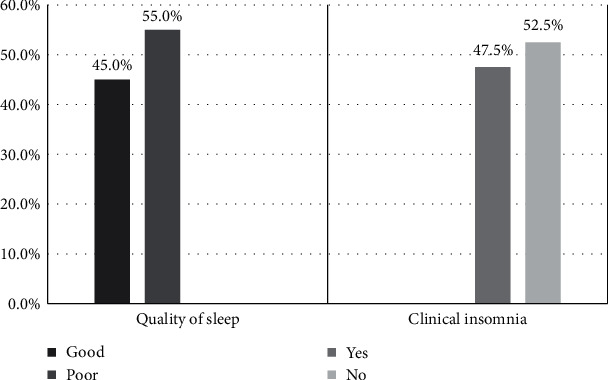
Distribution of clinical insomnia and quality of sleep.

On sleep quality using the PSQI scores, 55.0% of patients were found to suffer from poor quality of sleep (Figure 1). The mean PSQI score was 7.1 ± 5.1 (Table 1). 50.0% of patients suffered from comorbid diabetes and 63.6% from cardiovascular disease. Most patients had an advanced tumor grade ≥ 2 (81.8%) and lymph node invasion (72.8%) but no distant metastasis (90.9%). Most patients received chemotherapy (68.2%) and none received radiotherapy (Table 2).

On insomnia severity, the prevalence of clinical insomnia among patients with pancreatic cancer who had recently undergone surgery was 47.2% (Figure 1). The mean ISI score was 7.2 ± 6.9 (Table 1). Out of the 19 patients with clinical insomnia, 63.2% suffered from mild insomnia with an ISI score of 8–14 while the other 36.8% suffered from moderate to severe insomnia with an ISI score of ≥15. Patient age, alcohol consumption, smoking, or comorbid conditions were not significantly different between the mild insomnia and the moderate to severe insomnia groups. Additionally, the tumor grade and stage did not appear to statistically influence the severity of insomnia. The nature, severity, and impact of insomnia were significantly affected by adjuvant chemotherapy, with 50.0% of patients with mild insomnia receiving adjuvant chemotherapy versus 85.7% of patients with moderate to severe insomnia (Table 3).

**Figure 2:**
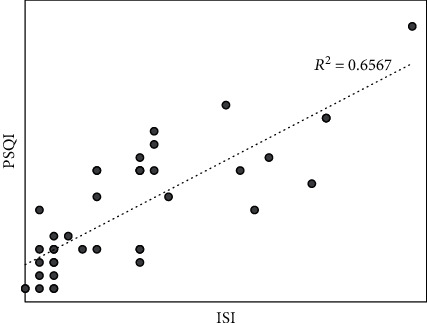
Scatterplot of ISI and PSQI scores.

On bivariate correlation analysis, there was a strong positive correlation between the sleep quality and insomnia scores (*r* = 0.78, *p* < 0.01) (Figure 2).

## 4. Discussion

Sleep disturbances severely affect cancer patients, with some studies reporting a prevalence of up to 70% [11]. Pancreatic cancer patients, especially in an immediate postoperative period, frequently report sleep disturbances. However, the prevalence of insomnia has not well been established in this population, as compared to other symptoms such as pain or anorexia.

In 1931, Yaskin reported depression and insomnia as presenting symptoms of pancreatic cancer [12]. More recently, Boyd et al. [13] considered depression as a prodrome for the disease itself. However, no studies focus solely on insomnia disorder. A study by Holly et al. showed that altered ability of sleep was reported 3 times more in pancreatic cancer patients than in the general population [14]. Pancreatic cancer is defined as a disease of the elderly, with >90% of patients aged above 55 years old, more common in men than in women (5.5 age-standardized rate in men vs. 4 in women) [15].

Our study retrospectively explored the occurrence and severity of insomnia in pancreatic cancer patients treated with pancreaticoduodenectomy in a single tertiary care center in Lebanon. Our population had similar patient characteristics to reported results by the IARC and GCO in the GLOBOCAN 2018 [16].

Pezzilli et al. showed impairment of sleep in patients with pancreatic cancer postpancreaticoduodenectomy [17]. Our results are consistent with those of Pezzilli et al., showing existence of sleep disturbances after surgery. Halloran et al. [18] found that pancreatic exocrine insufficiency after partial pancreaticoduodenectomy for pancreatic malignancy was strongly associated with insomnia. Our results are consistent with those of Halloran showing that more than half of the patients who undergo pancreatic surgery for pancreatic cancer suffer from sleep disturbance.

Our data additionally suggests that chemotherapy plays a role in the severity and impact of insomnia, as patients receiving chemotherapy suffer from more severe insomnia as compared to patients who do not. These results are consistent with previous studies where higher rates of sleep disturbances were observed in patients undergoing chemotherapy for breast cancer [19].

To our knowledge, this is the first study of its kind in the Middle East to evaluate prevalence and severity of insomnia in this subset of population using validated tools. Results of this study showed that pancreaticoduodenectomy is associated with a high prevalence of clinically important insomnia, thus highlighting the need for prospective studies conducted at a larger scale to understand the effect of sleep disturbances on a cancer patient's quality of life and response to treatment.

Limitations include our small sample size and the lack of additional knowledge on the patients' sleeping habits. Despite these limitations in this relatively small cohort of postoperative pancreatic cancer patients, insomnia and poor quality of sleep were shown to be highly prevalent.

Sleep disturbances in pancreatic cancer patients who have undergone pancreaticoduodenectomy are multifactorial. Postoperative recovery, chemotherapy, and the psychological impact of a cancer diagnosis have been implicated [17, 20]. A systematic review by Otte et al. has shown that although symptoms of poor sleep in cancer patients are being addressed by their healthcare providers, the primary focus shifts away from characterizing the underlying sleep disorders [21]. Thus, focused screening tools aimed at diagnosing the underlying pathology of sleep disturbances are needed in addition to more data on the prevalence and incidence of sleep disturbances in patients diagnosed with cancer. This ensures a problem-focused solution, especially in our region of the world where access to extensive mental health therapy may be limited.

## 5. Conclusion

Sleep disturbances after pancreatic cancer surgery have not been thoroughly evaluated in the literature. Our study highlighted the high prevalence of disordered sleeping in this category of patients and identified its impact on their daily lives. Additionally, chemotherapy plays a role in the severity and impact of insomnia. Treating physicians and surgeons should recognize and routinely screen for sleep disorders in postoperative pancreatic cancer patients, in order to alleviate some of the burden on their mental well-being and improve their quality of life. Further studies and tools are needed to better characterize and treat sleep disturbances in pancreatic cancer patients.

## Figures and Tables

**Table 1:** Patient characteristics.

Variable			Value
Age (years), mean ± SD			66.4 ± 10.1
Gender (%)	Male		27 (67.5%)
	Female		13 (32.5%)
BMI (kg/m^2^), mean ± SD			25.7 ± 5.4
Smoking, *n* (%)			18 (45.0%)
Alcohol consumption, *n* (%)			8 (20.0%)
Caffeine intake, *n* (%)			18 (45.0%)
Physical activity, *n* (%)			22 (55.0%)
Comorbidities, *n* (%)	Diabetes		17 (42.5%)
	COPD		2 (5.0%)
	CVD		21 (52.5%)
	CKD		4 (10.0%)
Tumor characteristics, *n* (%)	Grade	1	7 (17.5%)
		2	24 (60.0%)
		3	8 (20.0%)
		4	1 (2.5%)
	T stage	1	7 (17.5%)
		2	24 (60.0%)
		3	8 (20.0%)
		4	1 (2.5%)
	N stage	0	16 (40.0%)
		1	16 (40.0%)
		2	8 (20.0%)
	M stage	0	14 (35.0%)
		1	26 (65.0%)
Tumor size (cm), mean ± SD			2.7 ± 1.2
Treatment regimen, *n* (%)	Adjuvant chemotherapy		22 (55.0%)
	Neoadjuvant chemotherapy		2 (5.0%)
	Adjuvant radiotherapy		1 (2.5%)
	Neoadjuvant radiotherapy		0 (0.0%)
	No chemotherapy or radiotherapy		15 (37.5%)
All-cause mortality, *n* (%)			5 (12.5%)
PSQI score, mean ± SD			7.1 ± 5.1
ISI score, mean ± SD			7.2 ± 6.9

BMI: body mass index; COPD: chronic obstructive pulmonary disease; CVD: cardiovascular disease; CKD: chronic kidney disease; PSQI: Pittsburgh Sleep Quality Index; ISI: Insomnia Severity Index.

**Table 2:** Quality of sleep in patients postpancreaticoduodenectomy.

Variable			PSQI score > 5 (*n* = 22)
Age, mean ± SD			65.4 ± 11.0
Gender, *n* (%)	Male		15 (65.4%)
	Female		7 (27.3%)
BMI, mean ± SD			25.5 ± 5.7
Smoking, *n* (%)			6 (27.3%)
Alcohol consumption, *n* (%)			4 (18.2%)
Caffeine intake, *n* (%)			9 (40.9%)
Physical activity, *n* (%)			11 (50.0%)
Comorbidities, *n* (%)	Diabetes		11 (50.0%)
	COPD		1 (4.5%)
	CVD		14 (63.6%)
	CKD		2 (9.1%)
Tumor characteristics, *n* (%)	Grade	1	3 (13.6%)
		2	14 (63.6%)
		3	4 (18.2%)
		4	0
	T stage	1	4 (18.2%)
		2	12 (54.5%)
		3	6 (37.3%)
		4	0
	N stage	0	6 (27.3%)
		1	10 (45.5%)
		2	6 (27.3%)
	M stage	0	20 (90.9%)
		1	2 (9.1%)
Tumor size, mean ± SD			2.9 ± 1.4
Treatment regimen, *n* (%)	Adjuvant chemotherapy		13 (59.1%)
	Neoadjuvant chemotherapy		2 (9.1%)
	Adjuvant radiotherapy		0
	Neoadjuvant radiotherapy		0
	No chemotherapy or radiotherapy		7 (31.8%)
All-cause mortality, *n* (%)			2 (11.1%)

BMI: body mass index; COPD: chronic obstructive pulmonary disease; CVD: cardiovascular disease; CKD: chronic kidney disease; PSQI: Pittsburgh Sleep Quality Index.

**Table 3:** Prevalence of mild insomnia and moderate to severe insomnia in patients postpancreaticoduodenectomy.

Variable			ISI score 8–14 (*n* = 12)	ISI score ≥ 15 (*n* = 7)
Age, mean ± SD			68.6 ± 9.5	61.7 ± 13.2
Gender, *n* (%)	Male		8 (66.7%)	5 (71.4%)
	Female		4 (33.3%)	2 (28.6%)
BMI, mean ± SD			27.0 ± 6.4	24.4 ± 4.1
Smoking, *n* (%)			2 (16.7%)	3 (42.9%)
Alcohol consumption, *n* (%)			2 (16.7%)	3 (42.9%)
Caffeine intake, *n* (%)			5 (41.7%)	5 (71.4%)
Physical activity, *n* (%)			8 (66.7%)	5 (71.4%)
Comorbidities, *n* (%)	Diabetes		5 (41.7%)	3 (42.9%)
	COPD		0	0 (4.5%)
	CVD		6 (50.0%)	4 (57.1%)
	CKD		0	1 (14.3%)
Tumor characteristics, *n* (%)	Grade	1	1 (8.3%)	1 (14.3%)
		2	7 (58.3%)	6 (85.7%)
		3	4 (33.3%)	0
		4	0	0
	T stage	1	0	2 (28.6%)
		2	9 (75.0%)	4 (57.1%)
		3	3 (25.0%)	1 (34.3%)
		4	0	0
	N stage	0	3 (25.0%)	2 (28.6%)
		1	5 (41.7%)	4 (57.1%)
		2	4 (33.3%)	1 (14.3%)
	M stage	0	11 (91.7%)	7 (100.0%)
		1	1 (8.3%)	0
Tumor size, mean ± SD			2.8 ± 1.1	2.8 ± 1.4
Treatment regimen, *n* (%)	Adjuvant chemotherapy^∗^		6 (50.0%)	6 (85.7%)
	Neoadjuvant chemotherapy		0	1 (14.3%)
	Adjuvant radiotherapy		0	0
	Neoadjuvant radiotherapy		0	0
	No chemotherapy or radiotherapy		6 (50.0%)	0
All-cause mortality, *n* (%)			1 (8.3%)	0

BMI: body mass index; COPD: chronic obstructive pulmonary disease; CVD: cardiovascular disease; CKD: chronic kidney disease; ISI: Insomnia Severity Index; PSQI: Pittsburgh Sleep Quality Index. ^∗^Significance at *p* < 0.05; ^∗∗^significance at *p* < 0.01.

## Data Availability

Data is available upon request from the Department of General Surgery at the American University of Beirut Medical Center.

## References

[1] Beckmann J., Elbe A.-M. (2015). *Sport Psychological Interventions in Competitive Sports*.

[2] Fullagar H. H. K., Skorski S., Duffield R., Hammes D., Coutts A. J., Meyer T. (2015). Sleep and athletic performance: the effects of sleep loss on exercise performance, and physiological and cognitive responses to exercise. *Sports medicine*.

[3] Association ASD (1997). *International Classification of Sleep Disorders*.

[4] American Psychiatric Association (2013). *Diagnostic and Statistical Manual of Mental Disorders (DSM-5®)*.

[5] Roth T. (2007). Insomnia: definition, prevalence, etiology, and consequences. *Journal of clinical sleep medicine*.

[6] Bhaskar S., Hemavathy D., Prasad S. (2016). Prevalence of chronic insomnia in adult patients and its correlation with medical comorbidities. *Journal of family medicine and primary care*.

[7] Savard J., Morin C. M. (2001). Insomnia in the context of cancer: a review of a neglected problem. *Journal of clinical oncology*.

[8] Su X., Wang D.-X. (2018). Improve postoperative sleep: what can we do?. *Current opinion in anaesthesiology*.

[9] Buysse D. J., Reynolds C. F., Monk T. H., Berman S. R., Kupfer D. J. (1989). The Pittsburgh Sleep Quality Index: a new instrument for psychiatric practice and research. *Psychiatry Research*.

[10] Buysse D. J., Ancoli-Israel S., Edinger J. D., Lichstein K. L., Morin C. M. (2006). Recommendations for a standard research assessment of insomnia. *Sleep*.

[11] Fiorentino L., Ancoli-Israel S. (2007). Sleep dysfunction in patients with cancer. *Current treatment options in neurology*.

[12] Yaskin J. C. (1931). Nervous symptoms as earliest manifestations of carcinoma of the pancreas. *Journal of the American Medical Association*.

[13] Boyd A. D., Brown D., Henrickson C. (2012). Screening for depression, sleep-related disturbances, and anxiety in patients with adenocarcinoma of the pancreas: a preliminary study. *The Scientific World Journal*.

[14] Holly E. A., Chaliha I., Bracci P. M., Gautam M. (2004). Signs and symptoms of pancreatic cancer: a population-based case-control study in the San Francisco Bay area. *Clinical Gastroenterology and Hepatology*.

[15] Bindra B. S., Kaur H., Portillo S., Emiloju O., de de Jesus K. G. (2019). B-cell prolymphocytic leukemia: case report and challenges on a diagnostic and therapeutic forefront. *Cureus*.

[16] Bray F., Ferlay J., Soerjomataram I., Siegel R. L., Torre L. A., Jemal A. (2018). Global cancer statistics 2018: GLOBOCAN estimates of incidence and mortality worldwide for 36 cancers in 185 countries. *CA: a cancer journal for clinicians*.

[17] Pezzilli R., Falconi M., Zerbi A. (2011). Clinical and patient-reported outcomes after pancreatoduodenectomy for different diseases: a follow-up study. *Pancreas*.

[18] Halloran C. M., Cox T. F., Chauhan S. (2011). Partial pancreatic resection for pancreatic malignancy is associated with sustained pancreatic exocrine failure and reduced quality of life: a prospective study. *Pancreatology*.

[19] Fakih R., Rahal M., Hilal L. (2018). Prevalence and severity of sleep disturbances among patients with early breast cancer. *Indian journal of palliative care*.

[20] George M., Elias A., Shafiei M. (2015). Insomnia in cancer-associations and implications. *Asian Pacific Journal of Cancer Prevention*.

[21] Otte J. L., Carpenter J. S., Manchanda S. (2015). Systematic review of sleep disorders in cancer patients: can the prevalence of sleep disorders be ascertained?. *Cancer medicine*.

